# Early leaf senescence observed in the *pen1pen3* double mutant is not dependent on HIR2, a protein that physically interacts with PEN1.

**DOI:** 10.17912/micropub.biology.002392

**Published:** 2026-09-04

**Authors:** Tina A. Nguyen, David A. Soriano, Judy A. Brusslan

**Affiliations:** 1 Neurology and Behavior, UC Irvine, Irvine, CA, United States; 2 Medicine, University of Toledo, Toledo, OH, United States; 3 Biological Sciences, California State University, Long Beach, Long Beach, CA, United States

## Abstract

The *pen1pen3* double mutant displays early leaf senescence that depends on salicylic acid biosynthesis, a phytohormone associated with defense and leaf senescence. PEN1, a Qa SNARE, physically interacts with HIR2, a protein that tethers immune receptors to plasma membrane nanodomains. We hypothesized that PEN1 binding to HIR2 may prevent immune receptor localization. Consistent with this hypothesis is the expectation that *pen1pen3hir2* mutants would show reduced early leaf senescence when compared to *pen1pen3* . We constructed the triple mutant, quantified leaf senescence, and observed early leaf senescence that was not significantly different than *pen1pen3* . This result does not support our hypothesis.

**Figure 1. Analysis of the pen1pen3hir2 triple mutant:** Analysis of the *pen1pen3hir2* triple mutant. Panel A shows the confirmation of the *pen1pen3hir2* genotype. SALK_124393_LP/RP primers amplify the WT *HIR2* allele while *SALK* _ *1* 24393_RP/SALK_LBP primers amplify the *hir2* allele with the T-DNA insertion. Sequences of *pen1-1* and *pen3-1* are shown to the right and the mutant nucleotides are circled. Panel B shows the above-ground phenotypes of a representative plant from each genotype after seven weeks of growth. Panel C shows the numbers of leaves that were fully green, < 50% yellow, > 50% yellow and brown at the time of harvest (7 weeks). The percent of leaves that were < 50% yellow, > 50% yellow and brown were compared between the four genotypes in the graph below. Although not shown, comparisons between WT and *pen1pen3* and *pen1pen3hir2* as well as between *hir2* and *pen1pen3* and *pen1pen3hir2* were significant (****). Panel D shows the chlorophyll and *SGR1* gene expression quantities for each genotype. (p-values are as indicated: * < 0.05, ** < 0.01, *** < 0.005, **** < 0.001)

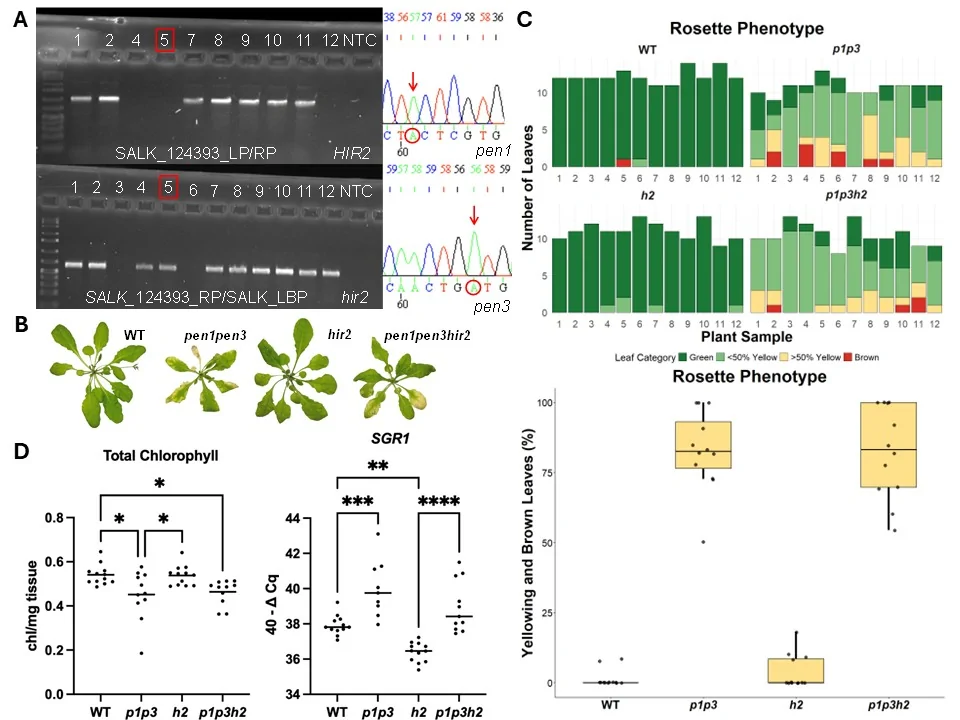



## Description

**Description**

In plants, leaf senescence is a process where older leaves turn yellow due to chlorophyll degradation as they export nutrients, primarily nitrogen, to support the growth of new tissues. The timing of leaf senescence is regulated by many mechanisms since both early and late leaf senescence are detrimental (Woo et al., 2019). Early leaf senescence does not allow adequate growth to support reproduction while late leaf senescence can increase the sink strength of the leaves, rendering them less capable of nutrient export.

PEN1/SYP121 (AT3G11820) is a syntaxin of plants that is the Qa component of a plasma membrane target-SNARE complex (PEN1-SNAP33) that docks transport vesicles via binding to vesicle SNAREs (VAMP 721/722) for vesicle fusion and exocytosis (Lipka et al., 2007; Bassham and Blatt, 2008). PEN1 is important for the localized thickening of the cell wall (papillae formation) that slows the penetration of fungal hyphae, one mechanism of nonhost resistance (Collins et al., 2003). *pen1* mutants were first identified based on faster fungal penetration. In addition, although *pen1* mutants displayed a normal phenotype in the absence of infection, they displayed a constitutive increase in the phytohormone salicylic acid (SA) (Zhang et al., 2007). SA is a positive regulator of pattern triggered immunity (PTI) and leaf senescence (Tian et al., 2025).

PEN3/PDR8 (AT1G59870) is an ABC transporter that carries the indole-derived antimicrobial molecule, camalexin, across the plasma membrane (He et al., 2019). *pen3* mutants were selected based on faster fungal penetration (Stein et al., 2006) and *pen3* mutants accumulate camalexin post-infection. *pen3* mutants also show leaf yellowing when grown under high light (HL) intensity (900 µmoles photons/m ^2^ /sec). The HL-induced chlorosis is dependent on SA and is not observed when SA synthesis is inhibited by mutation in isochorismate synthase1 ( *sid2* ).

We previously noted that *pen1pen3* double mutants displayed early leaf senescence when grown under lower light intensity (40 µmoles photons/m ^2^ /sec) (Crane et al., 2019). The plants grew normally for approximately six weeks, and then older leaves rapidly turned yellow. The early leaf senescence phenotype was reversed by the *sid2* mutation, demonstrating that the early leaf senescence was dependent on SA. These findings suggest that PEN1 and PEN3 have an additive effect on SA levels, with elevated SA levels leading to premature leaf senescence.

GFP-tagged PEN1 was shown to physically interact with Hypersensitive Induced Reaction2 (HIR2, AT3G01290) in microsomal fractions using immunoprecipitation followed by mass spectrometry (Fujiwara et al., 2014). HIR2 contains a stomatin/prohibitin/flotillin/HflK/C (SPFH) domain. There are four *HIR* paralogs in Arabidopsis and *HIR2* is strongly expressed in leaves. HIR2 localizes to membrane nanodomains via S-acylation (Weber et al., 2025; Daněk et al., 2026). HIR2 interacts with a wide variety of leucine-rich receptor protein kinases including FLS2 that mediates PTI and BAK1, a co-receptor for many immune receptors (Wu et al., 2025). Arabidopsis lines that overexpress *HIR2* show increased PTI (Qi et al., 2011). HIR2 is likely an assembly complex that tethers proteins to plasma membrane nanodomains. Importantly, SA levels were reduced by 70% in tobacco plants with silenced *NbHIR3* expression and transient overexpression of *NbHIR3* increased SA levels (Li et al., 2019). In addition, a cell death inducing protein (SsXyl2) from a necrotrophic fungal pathogen interacts with HIR2 in tobacco ( *Nicotiana benthamiana* ) at the plasma membrane to promote cell death activity (Wang et al., 2024). *Nbhir2* knockout mutants showed a reduced hypersensitive response and transient expression of both *SsXyl2* and *NbHIR2* resulted in larger areas of cell death.

We asked whether the early senescence phenotype of *pen1pen3* was dependent on HIR2. Was it possible that PEN1 interaction with HIR2 prevents HIR2-induced PTI such that a *pen1* mutant allows unrestrained HIR2 activity and early leaf senescence? To address this question, we produced *pen1pen3hir2* mutants and quantified their leaf senescence phenotypes. We hypothesized that *pen1pen3hir2* mutants would display a less severe early leaf senescence phenotype when compared to *pen1pen3* .

A T-DNA insertion for *hir2-2* (SALK_124393, named *hir2* in this study) was crossed to *pen1-1pen3-1* . One triple mutant (line 5, panel A) was selected from the F _3_ generation as being homozygous for a T-DNA insertion disrupting the fourth exon of the *HIR2* open reading frame (Weber et al., 2025) and for two separate point mutations that produce nonsense ( *pen1-1* , Collins et al., 2003) and missense ( *pen3-1* , Stein et al., 2006) null mutations (panel A). WT, *pen1pen3* , *hir2* and *pen1pen3hir2* plants were grown under a 20:4 long day light cycle (40 µmoles photons/m ^2^ /sec) under nutrient replete conditions (n=12 per genotype). These conditions minimize abiotic stress, which can promote leaf senescence. After six weeks of growth, both *pen1pen3* and *pen1pen3hir2* started to show yellowing of mature rosette leaves while WT and *hir2* remained green. At seven weeks, plants were harvested, and panel B shows representative plants of each genotype. Initially, the rosette leaves for each plant were characterized as green, <50% yellow, > 50% yellow or brown. Panel C shows that WT and *hir2* individuals had nearly all green rosette leaves while nearly all *pen1pen3* and *pen1pen3hir2* rosette leaves displayed at least some yellowing. The percentage of leaves with <50% yellow, > 50% yellow and brown was compared between *pen1pen3* and *pen1pen3hir2* and no significant difference was observed (One-way ANOVA, Tukey's HSD correction for multiple comparisons, p-value = 0.999). Similarly, no significant difference was observed between WT and *hir2* (p-value 0.952).

After the initial rosette leaf observations, leaf 6 from each individual plant was weighed and stored at -80 ^o^ C prior to chlorophyll quantitation (Panel D). Compared to WT, chlorophyll levels were lower in *pen1pen3* and in *pen1pen3hir2* . There was no significant difference between *pen1pen3* and *pen1pen3hir2* or between WT and *hir2* . The difference between *hir2* and *pen1pen3hir2* was close to significance (One-way ANOVA, Tukey's HSD correction for multiple comparisons, p-value = 0.0661).

Leaves 4 and 5 were harvested together from each individual plant and expression of *SGR1* , a senescence-associated gene, was measured using real-time qPCR with *ACT2* as the reference transcript (Panel D). *SGR1* expression was significantly higher in *pen1pen3* and in *pen1pen3hir2* compared to WT and *hir2* , respectively. *SGR1* expression was not significantly different when *pen1pen3* and *pen1pen3hir2* were compared (One-way ANOVA, Tukey's HSD correction for multiple comparisons, p-value 0.1727). A decrease in *SGR1* expression was noted between *hir2* when compared to WT, suggesting HIR2 may have an independent positive role in leaf senescence; however, delayed leaf senescence was not seen when chlorophyll was quantified. Both *pen1pen3* and *pen1pen3hir2* exhibited similar early leaf senescence indicating that *hir2* does not reverse the leaf senescence symptoms. These data do not support our hypothesis, and the proposed role for PEN1 in sequestering HIR2 to prevent early leaf senescence is not supported by our experimental evidence.

Although PEN1 has been shown to interact with HIR2, our findings demonstrate this interaction is not playing a role in early leaf senescence conferred by *pen1pen3* . Elevated SA contributes to early leaf senescence in *pen1pen3,* and lower SA was noted in *NbHIR3* -silenced tobacco lines; however, the *hir2* mutation in Arabidopsis did not alleviate early leaf senescence. One caveat is the three additional Arabidopsis *HIR* paralogs, although PEN1 interaction with these proteins was not observed. Regardless, this potential genetic redundancy may complicate analysis. In the future, quadruple mutants ( *pen1pen3hir2hir3* ) including the paralog with the next highest expression level in leaves, *HIR3* (AT1G69840) *,* can be compared to *pen1pen3* . In addition, SA levels in the *hir2-2* mutant should be quantified.

## Methods

*hir2-2* (AT3G01290, SALK_124393) is a T-DNA insertion disrupting the 4 ^th^ exon in the open reading frame and is a knockout mutant (Weber et al., 2025). *pen1-1* (AT3G11820, TAIR Accession Polymorphism:1010235864) is a nonsense mutation, C to T at chromosome 3 position 3730649 (A on reverse strand of panel A, Collins et al., 2003). *pen3-1* (AT1G59870, TAIR Accession Polymorphism:1010235837) is a missense G354D, G to A at chromosome 1 position 22036079 (Stein et al., 2006). Mutant lines were obtained from the Arabidopsis Biological Resource Center (Columbus, OH). Chlorophyll quantitation, RNA extraction, cDNA synthesis and real-time qPCR have been described (Zimmerman et al., 2024). One-way ANOVA with Tukey's HSD correction was used for multiple comparisons (GraphPad Prism).

## Reagents

Primers used in this study:

**HIR2:**

SALK_124393_LP: CACTCCTGCGACTTTCTTCTC

SALK_124393_RP: ACCATGTCCAACACATCCTTC

SALK_LBP: GGATTTTGCCGATTTCGGAACC

**PEN1**

PEN1_643_F: CAGAGGTCCTGGTTCGATCG

PEN1_643_R: CTTCCCTTCTCCGCCATTGT

pen1-1_643_Seq: GCCTTCTTCAACGCGACTCC

**PEN3**

PEN3_721_F: GTGAGTCCAACTCTCGCCTT

PEN3_721_R: AAGGTCTGTTCGGGTTCACC

pen3-1_721_Seq: CCGTGTAACAGATTTTGGGGC

**qPCR**

ACT2_F: GCGACTTGACAGAGAAGAAC

ACT2_R: GAAAGAGCGGAAGAAGATGAG

SGR1_F: TGGGCAAATAGGCTATACCG

SGR1_R: CCACCGCTTATGTGACAATG

## References

[R1] Bassham Diane C., Blatt Michael R. (2008). SNAREs: Cogs and Coordinators in Signaling and Development. Plant Physiology.

[R2] Collins NC, Thordal Christensen H, Lipka V, Bau S, Kombrink E, Qiu JL, et al., Schulze Lefert P. 2003. SNARE-protein-meidated disease resistance at the plant cell wall. Nature. 425: 973. 10.1038/nature02076 14586469

[R3] Crane Renee A., Cardénas Valdez Marielle, Castaneda Nelly, Jackson Charidan L., Riley Ciairra J., Mostafa Islam, Kong Wenwen, Chhajed Shweta, Chen Sixue, Brusslan Judy A. (2019). Negative Regulation of Age-Related Developmental Leaf Senescence by the IAOx Pathway, PEN1, and PEN3. Frontiers in Plant Science.

[R4] Daněk Michal, Hdedeh Omar, Amo Jesús, Boutet Jessica, Neubergerová Michaela, Safi Héla, Abuzeineh Anas, Martín‐Barranco Amanda, Fiche Jean‐Bernard, Mercier Caroline, Dumortier Baldwin, Krouk Gabriel, Nollmann Marcelo, Pleskot Roman, Boutté Yohann, Santoni Véronique, Mongrand Sébastien, Martinière Alexandre, Zelazny Enric (2026). Mechanisms controlling the plasma membrane targeting and the nanodomain organization of the plant SPFH protein HIR2. The Plant Journal.

[R5] Fujiwara Masayuki, Uemura Tomohiro, Ebine Kazuo, Nishimori Yuka, Ueda Takashi, Nakano Akihiko, Sato Masa H., Fukao Yoichiro (2014). Interactomics of Qa-SNARE in Arabidopsis thaliana. Plant and Cell Physiology.

[R6] He Yunxia, Xu Juan, Wang Xiaoyang, He Xiaomeng, Wang Yangxiayu, Zhou Jinggeng, Zhang Shuqun, Meng Xiangzong (2019). The Arabidopsis Pleiotropic Drug Resistance Transporters PEN3 and PDR12 Mediate Camalexin Secretion for Resistance to
*Botrytis cinerea*. The Plant Cell.

[R7] Li Saisai, Zhao Jinping, Zhai Yushan, Yuan Quan, Zhang Hehong, Wu Xinyang, Lu Yuwen, Peng Jiejun, Sun Zongtao, Lin Lin, Zheng Hongying, Chen Jianping, Yan Fei (2019). The
*hypersensitive induced reaction 3*
(
*
HIR3
*
) gene contributes to plant basal resistance via an
*
EDS1
*
and salicylic acid‐dependent pathway. The Plant Journal.

[R8] Lipka Volker, Kwon Chian, Panstruga Ralph (2007). SNARE-Ware: The Role of SNARE-Domain Proteins in Plant Biology. Annual Review of Cell and Developmental Biology.

[R9] Qi Yiping, Tsuda Kenichi, Nguyen Le V., Wang Xia, Lin Jinshan, Murphy Angus S., Glazebrook Jane, Thordal-Christensen Hans, Katagiri Fumiaki (2011). Physical Association of Arabidopsis Hypersensitive Induced Reaction Proteins (HIRs) with the Immune Receptor RPS2. Journal of Biological Chemistry.

[R10] Stein Mónica, Dittgen Jan, Sánchez-Rodríguez Clara, Hou Bi-Huei, Molina Antonio, Schulze-Lefert Paul, Lipka Volker, Somerville Shauna (2006). *Arabidopsis*
PEN3/PDR8, an ATP Binding Cassette Transporter, Contributes to Nonhost Resistance to Inappropriate Pathogens That Enter by Direct Penetration. The Plant Cell.

[R11] Tian Hainan, Xu Lu, Li Xin, Zhang Yuelin (2024). Salicylic acid: The roles in plant immunity and crosstalk with other hormones. Journal of Integrative Plant Biology.

[R12] Wang Pei, Wang Yabo, Hu Yawen, Chen Ziyang, Han Lili, Zhu Wenjun, Tian Binnian, Fang Anfei, Yang Yuheng, Bi Chaowei, Yu Yang (2023). Plant hypersensitive induced reaction protein facilitates cell death induced by secreted xylanase associated with the pathogenicity of
*Sclerotinia sclerotiorum*. The Plant Journal.

